# Daily dataset on temperature and relative humidity in two traditional Basque architectural models located in Lea river valley

**DOI:** 10.1016/j.dib.2019.104940

**Published:** 2019-12-05

**Authors:** Matxalen Etxebarria Mallea, Lauren Etxepare Igiñiz, Margarita de Luxán García de Diego

**Affiliations:** a Department of Architecture, University of the Basque Country (UPV/EHU), Oñati Plaza 2, 20018, Donostia-San Sebastian, Spain; b Technical University of Madrid (UPM), Spain

**Keywords:** Indoor hygrothermal monitoring, Indoor hygrothermal simulation, Traditional architecture

## Abstract

This data article compares two daily datasets of temperature and relative humidity in two traditional Basque architectural models (B1, B2) located in Lea river valley (Basque Country, Northern Spain). The datasets are drawn from two different approaches to data collection, computer-based simulation and an in-situ monitoring. For this purpose, machine-learning models of the two traditionally constructed buildings were developed and simulated according to annual periods under their SWEC reference climate. These data were compared to the datasets derived from the indoor monitoring campaign, in which hygrothermal variables in two thermal zones of each building (B1_Z1, B1_Z2; B2_Z1, B2_Z2) were measured between September 2018 and September 2019. Accordingly, outdoor climate data were acquired from the nearest weather station (Iruzubieta, Ziortza-Bolibar).

**Table:** Specifications Table

Subject	Construction and architecture
Specific subject area	Building physics: outdoor and indoor environments monitoring and simulating
Type of data	Tables and figures
How data were acquired	Data were acquired in two buildings (B1, B2) with regard to two comparable approaches: machine learning models' hygrothermal data (EnergyPlus 8.6 simulation tool through DesignBuilder v.5.0.1.024 interface) vs indoor monitoring campaign through temperature and relative humidity sensors (SenNet DR-30-24 datalogger and SenNet DL THL-I measuring devices)
Data format	Raw, analyzed and filtered
Parameters for data collection	The selected traditionally constructed buildings had to represent different construction periods, belong to different construction types, hence, describe different thermal envelope construction characteristics, be located facing the southeast or south, be unoccupied (even if used at times) and non-thermally intervened. In addition, thermal zones facing the main solar façade were favoured for indoor temperature and relative humidity measurements and simulation.
Description of data collection	The measurement devices for the hygrothermal monitoring campaign were set up in two thermal zones of each building (ground floor: B1_Z1, B2_Z1; first floor: B1_Z2, B2_Z2), while the computer-based models considered all thermal zones of each building for the simulation process. However, from the hygrothermal dataset obtained, the ones corresponding to the monitored zones were extracted. Accordingly, local climate data from the nearest weather station was collected during the monitoring campaign.
Data source location	B1_building: Barrenetxea Farmhouse (43.255307, −2.583003) B2_building: Barrutieta Farmhouse (43.257857, −2.589525) Town/Region: Munitibar-Arbatzegi-Gerrikaitz (Basque Country) Country: Spain
Data accessibility	With the article
Related research article	M. Etxebarria Mallea, L. Etxepare Igiñiz, M. De Luxán García de Diego, Passive hygrothermal behaviour and indoor comfort concerning the construction evolution of the traditional Basque architectural model. Lea valley case study, Building and Environment 143 (2018) 496–512, https://doi.org/10.1016/j.buildenv.2018.06.041

**Table:** 

**Value of the Data** • The data show the indoor hygrothermal behaviour of the traditional Basque architectural model and describe traditional construction envelopes' thermal physics. • The data could be used as basis for further comparison between in-situ measurements and computer simulations theoretical data. • The data is valuable to construction project professionals and could also be suitable for heritage intervention regulations development, so that respectful and more efficient intervention criteria could be defined according to real feedback. • The data could be useful for researchers evaluating the variation of indoor hygrothermal environments in different outdoor conditions.

## 1 Data description

The data shown in this article provide additional and complementary data to the already published article « Passive hygrothermal behaviour and indoor comfort concerning the construction evolution of the traditional Basque architectural model. Lea valley case study» [1], and they describe both computer-simulation-based and in-situ monitoring hygrothermal data (temperature and relative humidity) on daily basis (Tables 1–4 of the supplementary file).

**Fig. 1:**
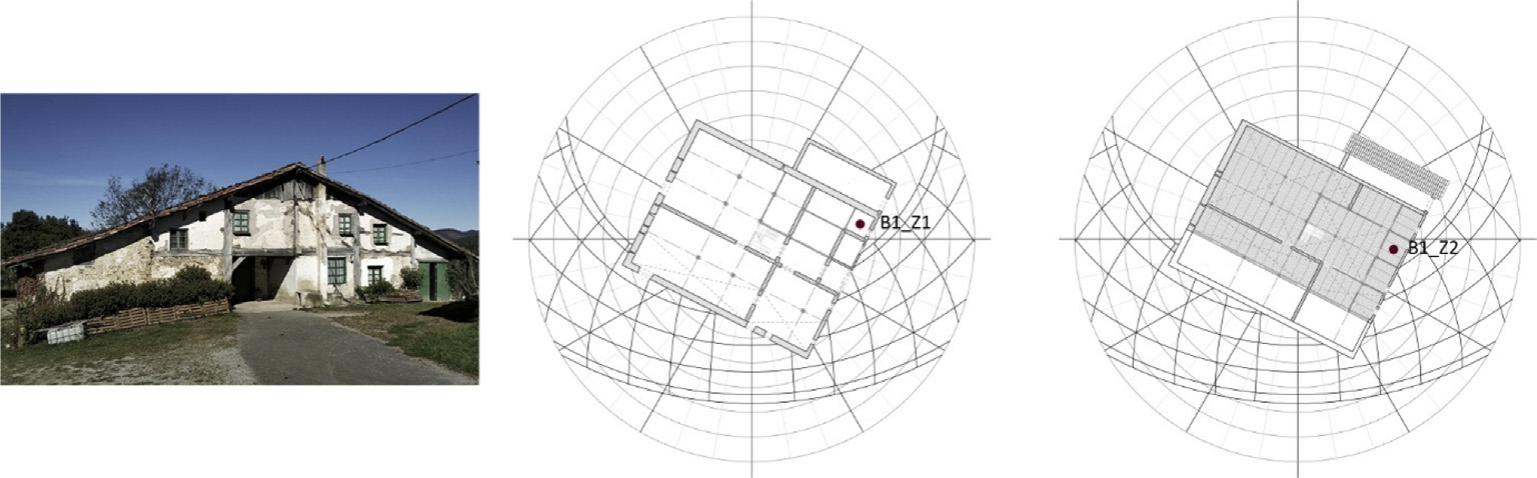
Barrenetxea farmhouse (B1). Main façade's current photo, ground floor and first floor. Own elaboration.

**Fig. 2:**
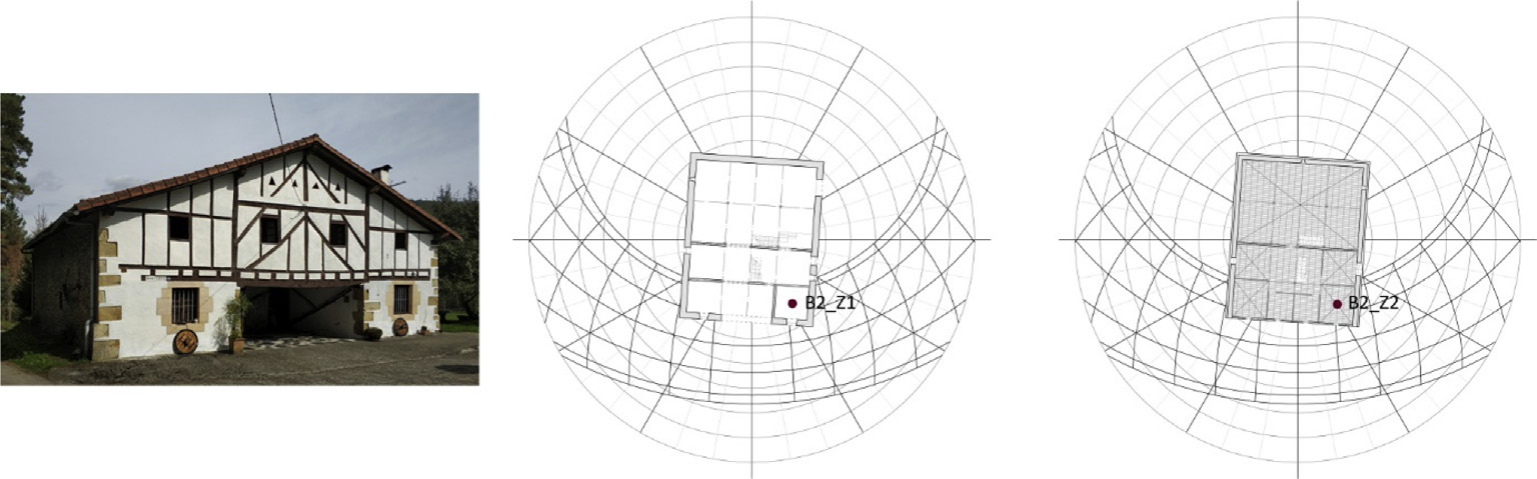
Barrutieta farmhouse (B2). Main façade's current photo, ground floor and first floor. Own elaboration.

The data correspond to the Renaissance model Barrenetxea (B1, Fig. 1, Tables 1–2) and to the Baroque model Barrutieta (B2, Fig. 2, Tables 3–4). The dataset describes the indoor hygrothermal behaviour of two thermal zones located in each traditional building, that is, B1_Z1, B1_Z2 in Barrenetxea and B2_Z1, B2_Z2, instead, in Barrutieta.

In addition to indoor data, the provided dataset includes outdoor climate data too; simulation data refer to San Sebastian-SWEC reference climate data [2], while local climate data correspond to the weather station located in Iruzubieta (Ziortza-Bolibar municipality, 10km far from the buildings), which belongs to the Basque Meteorology Agency-Euskalmet [3].

With regard to data comprehension, computer-based dataset illustrates completely passive buildings, that is, with no active energy generation systems switched on, while monitoring data, even if almost all describe passive states, vary between passive and active values. The active energy generation system used was a fireplace, which warmed the entire building up from its location in the monitored ground floor thermal zones (B1_Z1, B2_Z1).

## 2 Experimental design, materials, and methods

Machine-learning models were developed with EnergyPlus 8.6 simulation tool through DesignBuilder v.5.0.1.024 interface, evaluated a climatic annual period and followed the method described in the previously mentioned article [1].

Indoor temperature (T^a^) and relative humidity (HR) monitoring included a SenNet DR-30-24 datalogger and two SenNet DL THL-I measuring devices per building (one in each thermal zone), which automatically gathered data every 15 minutes. The data shown, however, describe daily basis.

The monitored and simulated thermal zones were two in each building:

• Barrenetxea farmhouse (B1, Fig. 1):
  − Ground floor thermal zone (B1_Z1): useful area of 6.24m^2^, sandstone masonry wall (e = 40cm), facing the southeast
  − First floor thermal zone (B1_Z2): useful area of 8.48m^2^, sandstone masonry + timber framework wall (e = 25cm), facing the southeast
• Barrutieta farmhouse (B2, Fig. 2):
  − Ground floor thermal zone (B2_Z1): useful area of 13.62m^2^, sandstone masonry wall (e = 70cm), facing the south
  − First floor thermal zone (B2_Z2): useful area of 16.56m^2^, sandstone masonry + timber framework wall (e = 18cm), facing the south

The monitoring campaign covered almost a year in each building, including unmeasured intervals due to technical failures:

• Barrenetxea farmhouse (B1): October 23rd 2018–September 30th 2019
• Barrutieta farmhouse (B2): September 21st 2018–August 31st 2019
